# Mastite granulomateuse idiopathique: à propos de 10 cas

**DOI:** 10.11604/pamj.2025.52.107.49391

**Published:** 2025-11-13

**Authors:** Valentine Séréna Ndong, Ghita Taki, Hicham Harmouche

**Affiliations:** 1 Service de Médecine Interne de l'Hôpital Universitaire Cheikh Zaïd, Université Internationale Abulcasis des Sciences de la Santé, Rabat, Maroc,; 2 Service de Médecine Interne du Centre Hospitalier Universitaire Ibn Sina Rabat, Faculté de Médecine, Université Mohamed V, Rabat, Maroc

**Keywords:** Mastite granulomateuse idiopathique, mastite granulomateuse, mastite lobulaire granulomateuse, traitement, Idiopathic granulomatous mastitis, granulomatous mastitis, granulomatous lobular mastitis, treatment

## Abstract

La mastite granulomateuse idiopathique (MGI) est une pathologie mammaire bénigne rare simulant les carcinomes mammaires. L'objectif de notre étude était de décrire le profil épidémiologique, clinique, thérapeutique et évolutif de la MGI. Il s'agissait d'une étude rétrospective descriptive sur les patientes suivies pour une MGI à l'Hôpital Cheikh Zaïd de 2019 à 2025. Au total, 10 patientes ont été incluses, correspondant à 83,33% de la MGI sur les 12 patientes suivies pour une mastite granulomateuse (MG). L'âge moyen de notre population était de 35,1 ans ± 4,65. Les antécédents étaient représentés par la grossesse et l'allaitement chez 100% avec une parité moyenne de 2,10 ± 0,568. La MGI était révélée par une mastodynie chez 4 cas (40%). L'examen clinique retrouvait un nodule mammaire ainsi qu'une atteinte unilatérale chez 100% des patientes. L'échographie mammaire et la mammographie étaient réalisées dans 100% des cas, de même que la biopsie en faveur d'une MG. Les corticoïdes étaient utilisés chez les 10 patientes en première intention avec une dose moyenne de 58mg/jour ± 14,8 avec une dose minimum de 20mg/jour de prednisone et une dose maximale de 80mg/jour avec une guérison complète chez 80% des patientes mais un taux de rechute de 20% avec un délai de 2 mois et de 7 mois après l'arrêt de la corticothérapie; 20% sous azathioprine et 40% étaient drainées. Il est essentiel de souligner l'importance de reconnaître la MGI dans la pratique clinique dont le traitement de première intention est basé sur la corticothérapie, tandis que les formes récurrentes peuvent nécessiter des immunosuppresseurs.

## Abstract (en)

Idiopathic granulomatous mastitis (IGM) is a rare benign breast pathology simulating breast carcinomas. The objective of our study was to describe the epidemiological, clinical, therapeutic, and evolutionary profile of IGM. This was a descriptive retrospective study of patients followed for IGM at Cheikh Zaid Hospital from 2019 to 2025. A total of 10 patients were included, corresponding to 83.33% of the 12 patients followed for granulomatous mastitis (GM). The mean age of our population was 35.1 years ± 4.65. The history was represented by pregnancy and breastfeeding in 100% with a mean parity of 2.10 ± 0.568. IGM was revealed by mastodynia in 4 cases (40%). Clinical examination found a breast nodule and unilateral involvement in 100% of patients. Breast ultrasound and mammography were performed in 100% of cases, as was biopsy in favor of MG. Corticosteroids were used in the 10 patients as first-line treatment with a mean dose of 58mg/day ± 14.8 with a minimum dose of 20mg/day of prednisone and a maximum dose of 80mg/day with plete healing in 80% of patients but a relapse rate of 20% with a delay of 2 months and 7 months after stopping corticosteroid therapy; 20% under azathioprine and 40% were drained. It is essential to emphasise the importance of recognising IMG in clinical practice, whose first-line treatment is based on corticosteroid therapy, while recurrent forms may require immunosuppressants.

## Introduction

La MGI est une maladie inflammatoire bénigne du sein [1], rare, simulant les carcinomes mammaires [2]. Elle se caractérise par la formation de granulomes non caséeux et de micro-abcès, limités aux lobules de la glande mammaire [3]. Le mécanisme pathologique demeure encore méconnu à ce jour [4]. À cause de la rareté de cette pathologie, le traitement de la MGI n'est pas consensuel [5]. Le but de ce travail est de décrire les caractéristiques épidémiologiques, cliniques, thérapeutiques et évolutives des MGI à l'Hôpital Cheikh Zaïd (HCZ).

## Méthodes

**Conception et contexte de l'étude:** cette étude rétrospective, observationnelle et descriptive a été menée à l'HCZ sur une période de 7 ans, de 2019 à 2025.

**Population étudiée et critère d'inclusion:** l'étude a porté sur les patientes suivies pour une MGI histologiquement confirmée avec un bilan étiologique négatif. Toutes les patientes ont été prises en charge au centre d'étude pendant la période définie.

**Critères d'exclusion:** les patientes ayant un diagnostic de mastite granulomateuse non idiopathique et donc spécifique étaient exclues, ainsi que les patientes ayant une MGI avec des dossiers non exploitables.

**Recueil et source des données:** la collecte des données a reçu l'approbation du comité d'éthique de la Faculté de Médecine de l'Université Internationale Abulcasis des Sciences et de la Santé de Rabat. Pour chaque cas de MGI, nous avons recueilli, à partir des dossiers cliniques, les paramètres suivants: les données socio-démographiques; les antécédents; les données cliniques et paracliniques; le traitement et l'évolution en précisant la guérison (les patientes étaient considérées comme guéries lorsqu'il n'y avait plus de masse palpable dans le sein et qu'il n'y avait plus de plaie résiduelle) ou les complications s'il y en a.

**Analyse statistique:** les données recueillies ont été saisies à l'aide du logiciel Microsoft Excel (Version 2311), puis analysées à l'aide du logiciel statistique JAMOVI (Version 2.6.26). Pour évaluer la distribution des variables quantitatives, le test de normalité de Shapiro-Wilk a été utilisé. Les variables qualitatives sont présentées à l'aide de tableaux de fréquences et/ou de représentations graphiques.

## Résultats

**Sur le plan épidémiologique:** nous avons colligé 10 patientes suivies pour une MGI à l'HCZ. Ces cas de MGI représentaient 83,33% (10/12) de l'ensemble des mastites granulomateuses (la MG tuberculeuse et la MG sarcoïdosique exclues). Nos 10 cas étaient des femmes (100%) et l'âge moyen de notre population était de 35,1 ans ± 4,65, avec une tranche d'âge comprise entre 26 et 41 ans.

**Les principaux antécédents:** la grossesse était retrouvée chez 100% des patientes avec une parité moyenne de 2,10 ± 0,56; l'allaitement chez 100% et la pilule contraceptive orale chez 20%.

**Figure 1:**
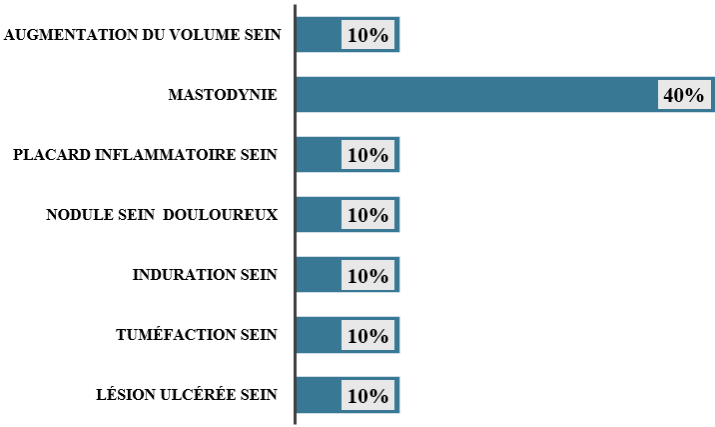
principaux symptômes de consultation et/ou d'hospitalisation [N=10, HCZ 2025]

**Données cliniques:** l'atteinte était unilatérale chez toutes les patientes (100%) avec une prédominance sur le sein droit dans 60% des cas, atteinte de 40% sur le sein gauche. Il n'y avait pas d'atteinte bilatérale. Les symptômes étaient dominés par la mastodynie dans 40% des cas, les autres symptômes sont représentés dans la Figure 1. L'examen clinique retrouvait chez nos 10 patientes (100%) un nodule mammaire; chez 4 cas une asymétrie mammaire et respectivement chez un cas une ulcération mammaire et une fistule mammaire. Les manifestations systémiques étaient représentées par un érythème noueux, des polyarthralgies, de la fièvre, une altération de l'état général chez 20% des patientes chacune.

**Figure 2:**
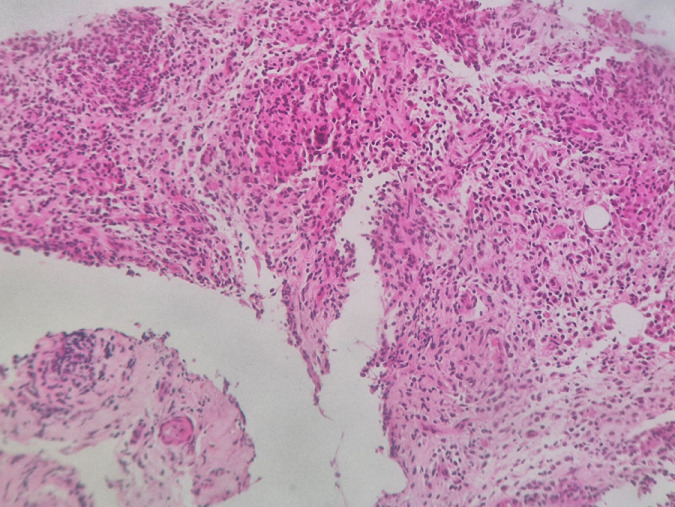
aspect histologique montrant la présence d'un granulome épithélio-giganto-cellulaire sans nécrose caséeuse [HCZ 2025]

**Données paracliniques**: l'échographie mammaire et la mammographie ont été réalisées dans 100% des cas, avec la mise en évidence d'une anomalie de densité à la mammographie chez 60% des patientes et d'une plage échogène à l'échographie mammaire dans 60% des cas. L'ectasie canalaire, la collection abcédée et les fistules mammaires étaient retrouvées respectivement chez 4 cas (40%) (Tableau 1). La biopsie avec étude histologique du matériel était réalisée chez les 10 patientes (100%). Elles étaient toutes en faveur d'une MG, caractérisée par la présence de granulomes inflammatoires non caséifiés dans les lobules contenant des lymphocytes, des plasmocytes, des éosinophiles, des histiocytes épithélioïdes et des cellules géantes multinucléées (Figure 2). La présence de la collection abcédée était retrouvée chez 40% de ces patientes. Les autres moyens diagnostiques utilisés étaient la tomographie par émission de positons scanner chez deux patientes (20%) avec la présence de 4 foyers actifs du sein droit chez l'une et de multiples foyers rétro-mammaires gauches hypermétaboliques chez l'autre. L'IRM mammaire était réalisée chez 2 cas (20%) et montrait respectivement de multiples collections du sein droit avec trajet fistuleux ainsi qu'une galactophorite avec des signes de mastite du sein gauche. Les autres examens complémentaires tels que la tomodensitométrie thoraco-abdomino-pelvienne ont été réalisés dans le but d'exclure un diagnostic différentiel. Les examens biologiques à la recherche d'une étiologie spécifique de la MG étaient tous soit normaux soit négatifs et comprenaient principalement: la culture des prélèvements biopsiques avec les colorations spécifiques; le GenXpert BK; l'ECA, le QuantiFERON, les sérologies virales (hépatite B, C et HIV), les AAN et les ANCA.

**Tableau 1:** principales anomalies retrouvées chez nos patientes à l'échographie mammaire et à la mammographie [HCZ 2025]

Données de l'échographie mammaire et de la mammographie	
1	Une asymétrie de densité dans les différents cadrans des seins atteints
2	Des anomalies de signaux avec plage hypoéchogène ou nodule hypoéchogène
3	Des collections abcédées du sein
4	Des trajets fistuleux du sein
5	Des adénomégalies axillaires
6	Des ectasies canalaires rétro aréolaires et galactophoriques
7	Des dystrophies fibrokystique sur fond mastosique

**Sur le plan thérapeutique:** 100% des patientes étaient traitées par les corticoïdes en première intention avec une dose moyenne de 58mg/jour ± 14,8 avec une dose minimale de 20mg/jour de prednisone et une dose maximale de 80mg/jour. La durée moyenne de la corticothérapie était de 14,7 ± 6,44 mois avec une médiane de 12 mois, un minimum de 9 mois et un maximum de 27 mois parmi les 6 (60%) chez qui la corticothérapie a été arrêtée. Quatre patientes (40%) avaient bénéficié d'une antibiothérapie probabiliste. Deux cas (20%) avaient reçu, en dehors de la corticothérapie, de la colchicine dans le cadre de l'érythème noueux et 4 (40%) avaient reçu un traitement d'épreuve initial antituberculeux. Quatre patientes (40%) avaient été drainées et aucune n'a bénéficié d'une chirurgie. Toutes les autres patientes (100%) étaient considérées comme guéries après la corticothérapie et 20% de ces patientes ont rechuté avec un délai de 2 mois et de 7 mois respectivement après l'arrêt de la corticothérapie. La corticothérapie était réintroduite chez ces patientes à la rechute. Chez ces dernières, (n=2; 20%), un autre immunosuppresseur a été ajouté: l'azathioprine. Il n'y avait pas de complication post-thérapeutique.

## Discussion

La mastite granulomateuse idiopathique a été décrite pour la première fois en 1972 chez cinq jeunes femmes en âge de procréer qui étaient opérées pour une suspicion de cancer du sein par Kessler et Wolloch [2]. La MG ne représente que, d'un point de vue épidémiologique, 0,44 à 1,6% des échantillons de biopsie mammaire selon les critères diagnostiques pathologiques et cytologiques selon les dernières données épidémiologiques [3]. La moyenne d'âge des patientes ayant une MGI dans notre série était de 35,1 ans ± 4,65, avec une tranche d'âge comprise entre 26 et 41 ans, se rapproche des données retrouvées dans la littérature notamment aux USA où Ringsted *et al*. et Thomas *et al*. rapportaient respectivement une moyenne d'âge de 32 ans et 39 ans dans leurs séries en 2021 et en 2023 [6,7]. Deux publications antérieures au Maroc par Fajri *et al*. [8] à Casablanca et Ennasser *et al*. [9] à Oujda sur des séries de 20 cas et de 4 cas ont rapporté respectivement une moyenne d'âge, de patientes suivies pour MGI, de 38,1 ans et 35,25 ans. Ça correspond à l'âge des femmes en âge de procréer principalement en per partum et post partum [4]. La prédominance féminine corrobore les résultats précédemment décrits par d'autres auteurs [6-9]. La grande fréquence de la MGI chez les femmes pourrait être expliquée par le fait que le sein est un organe qui est présent dans les deux sexes mais appartient principalement à la femme car il est atrophié et rudimentaire chez l'homme et chez la femme jusqu'à la puberté. La particularité faisant de cet organe une propriété chez la femme est qu'à la puberté, les glandes mammaires prennent un accroissement qui est en rapport avec le développement de l'appareil génital [10].

Le mécanisme pathologique de la MGI demeure encore méconnu et à ce jour, quatre hypothèses principales ont été évoquées pour élucider cette affection: l'origine auto-immune, le mécanisme auto-inflammatoire, la composante infectieuse et les troubles hormonaux [4]. De ce fait, sur le plan hormonal, l'allaitement et la parité étaient présents chez toutes nos patientes (100%) avec une parité moyenne de 2,10 ± 0,568. Cela rejoint les résultats rapportés par Ringsted *et al*. [6] qui ont retrouvé que 92 % des 28 patientes incluses dans leur série de cas avaient comme antécédents l'allaitement et la grossesse. En dehors des antécédents gestationnels, le rôle probable de l'hyperprolactinémie dans la pathogenèse de la MGI a été évoqué [11].

Les données cliniques retrouvées dans la littérature confirment les caractéristiques décrites dans notre série d'étude où la mastodynie était le symptôme prédominant dans 40% des cas et le nodule mammaire retrouvé dans 100% des cas et nous permettent ainsi d'identifier rapidement quels symptômes pourraient refléter leur fréquente association à la MGI ou à contrario leur moindre association dans la démarche diagnostique. L'inflammation axillaire, symptôme atypique de la MGI, a été présentée par Salih *et al*. [12] en décrivant un cas rare de MGI localisé dans le tissu mammaire accessoire axillaire chez une femme. Cela nous met en alerte sur la possibilité d'avoir une MGI au niveau de tous les tissus mammaires accessoires quelle que soit leur localisation.

Les principales manifestations systémiques rapportées dans notre étude de cas étaient dominées par l'érythème noueux, les polyarthralgies, la fièvre et l'altération de l'état général, respectivement chez 20% des patientes chacune. Ces données concordent avec celles retrouvées par Hamsho *et al*. ainsi que par Hashmi *et al*. qui rapportaient majoritairement l'érythème noueux [13,14], les arthralgies et/ou l'arthrite [13] et la fièvre [13,14]. Les autres manifestations moins fréquentes rapportées étaient l'épisclérite [13] et l'alopécie [7]. Ces manifestations pourraient témoigner de l'implication de mécanismes immunitaires, les reliant au granulome dans le sein caractéristique principale de la MGI, nécessitant une évaluation approfondie pour exclure d'éventuelles comorbidités auto-immunes.

Il est essentiel après l'étude histopathologique d'effectuer le bilan étiologique afin d'exclure les diagnostics différentiels de la MGI représentés dans le Tableau 2 [1]. Ces examens paracliniques comprennent la mise en culture des prélèvements avec la recherche spécifique de mycobactéries, recherche mycotique, la réalisation de colorations spécifiques à savoir Zielh Neelsen, BAAR, PAS, Grocott, l'ECA, les Ac anti nucléaires, les sérologies syphilitiques, virales hépatites B et C, VIH; la Rx thorax ou TDM thorax, les anticorps anti-nucléaires, les ANCA, l'IDR BK et la PCR recherche de BK sur prélèvement. Ils étaient soit négatifs, soit normaux chez nos 10 cas.

**Tableau 2:** diagnostics différentiels de la mastite granulomateuse idiopathique

Etiologies	-
Mastite granulomateuse neutrophilique cystique	-
Mastite infectieuse	Bactérienne: les espèces Cornybactéries en particulier *C. kroppenstedtii* et *C. tuberculostearium*, mastite périductale, abcès à actinomyces, mastite tuberculeuse, lèpre, maladie des griffes du chat. Fongique: histoplasmose, cryptococcose, coccidiomycose. Parasitaire: schistosomiase.
Auto immune	Vascularite artérite à cellules géantes artérite de Takayasu granulomateuse avec polyangéite granulomatose éosinophilique avec polyangéite maladie de Crohn sarcoïdose du sein
Granulomes à corps étrangers	Silicone Paraffine Béryllium
Mastite à IgG 4	-

Le traitement de la MGI n'est pas consensuel [5]. Proposés pour la première fois en 1980, les corticoïdes constituent le traitement de base de la MGI lorsque le diagnostic est posé [15]. Notre série de cas de MGI a donc été traitée en accord avec les données de la littérature par la corticothérapie orale, qui représente le traitement le plus répandu de la MGI. Malgré l'efficacité des corticoïdes en intra-lésionnels ou en systémiques, il existe des cas réfractaires ou qui rechutent chez qui l'attitude thérapeutique doit être réajustée. Alipour *et al*. [16], en Iran, ont publié un essai clinique démontrant que l'association du méthotrexate à une faible dose de corticoïde, dans les cas réfractaires, fait mieux que les corticoïdes seuls. L'azathioprine dans notre étude de cas s'avère une option thérapeutique alternative avec une efficacité chez nos 2 patientes chez qui il a été introduit. Lorsque les traitements de seconde ligne ne permettent pas d'obtenir la rémission, un traitement de troisième ligne s'impose. L'imiquimod est une option intéressante dans les cas réfractaires, comme l'ont démontré en 2024 Schön *et al*. [17] qui ont rapporté une guérison sous imiquimod chez 2 patientes suivies pour MGI traitées antérieurement par des AINS, de la colchicine, des corticoïdes à fortes doses et du méthotrexate. Les effets indésirables les plus courants de l'imiquimod sont les lésions cutanées, survenues chez les deux cas présentés par ces auteurs [17]. Les anti-TNF tels que le certolizumab pégol et l'adalimumab [18,19] ont été utilisés dans des rapports de cas, mais il faudrait des essais de grandes séries pour évaluer non seulement leur efficacité mais aussi l'innocuité de ces thérapeutiques dans le but de les inclure dans un protocole bien établi de prise en charge de la MGI. En dehors du traitement médical, la chirurgie est un autre moyen thérapeutique à proposer en 1^re^ intention ou chez les patients réfractaires ou rechuteurs, Tang *et al*. [20]. Notre étude a comme principale limite le nombre restreint de cas étudiés, mais nous permet néanmoins d'analyser les caractéristiques de notre population et d'en ressortir des informations cliniques et des implications pratiques importantes.

## Conclusion

La MGI est une pathologie mammaire bénigne rare qui affecte principalement les femmes en âge de procréer, particulièrement en per partum et post partum. Sa présentation clinique se caractérise par une masse mammaire unilatérale, douloureuse, associée à des signes inflammatoires locaux tels que l'érythème, l'induration ou la formation de fistules. Les symptômes systémiques chez certains patients peuvent compliquer le diagnostic différentiel avec d'autres pathologies inflammatoires ou infectieuses. La diversité des manifestations cliniques et des facteurs de risque souligne l'importance d'une approche diagnostique multidisciplinaire, combinant l'imagerie, la biopsie histologique et l'analyse microbiologique. Concernant la prise en charge, les stratégies thérapeutiques doivent être individualisées, incluant des traitements médicaux, notamment les corticostéroïdes et les immunosuppresseurs, ainsi que des interventions chirurgicales si nécessaire. Une meilleure reconnaissance et une gestion appropriée de la MGI sont essentielles pour améliorer le pronostic des patientes.

### Etat des connaissances sur le sujet

- La mastite granulomateuse idiopathique est une pathologie inflammatoire bénigne du sein rare, simulant les carcinomes mammaires;
- Le traitement de la mastite granulomateuse idiopathique n'est pas consensuel et repose sur la corticothérapie en première intention puis le méthotrexate en deuxième intention.

### Contribution de notre étude à la connaissance

- La mastite granulomateuse idiopathique représente 83,3% de toutes les mastites granulomateuses;
- L'évolution est souvent favorable sous corticothérapie avec un taux de guérison complet chez 80% des patientes et l'azathioprine peut être utilisée comme traitement de deuxième intention.
